# Suspected Locally Acquired Coccidioidomycosis in Human, Spokane, Washington, USA

**DOI:** 10.3201/eid2603.191536

**Published:** 2020-03

**Authors:** Hanna N. Oltean, Mark Springer, Jolene R. Bowers, Riley Barnes, George Reid, Michael Valentine, David M. Engelthaler, Mitsuru Toda, Orion Z. McCotter

**Affiliations:** Washington State Department of Health, Shoreline, Washington, USA (H.N. Oltean);; Spokane Regional Health District, Spokane, Washington, USA (M. Springer);; Translational Genomics Research Institute, Flagstaff, Arizona, USA (J.R. Bowers, R. Barnes, G. Reid, M. Valentine, D.M. Engelthaler);; Centers for Disease Control and Prevention, Atlanta, Georgia, USA (M. Toda, O.Z. McCotter)

**Keywords:** coccidioidomycosis, Coccidioides immitis, fungi, respiratory infections, canonical single-nucleotide polymorphisms, Spokane, Washington, USA

## Abstract

The full geographic range of coccidioidomycosis is unknown, although it is most likely expanding with environmental change. We report an apparently autochthonous coccidioidomycosis patient from Spokane, Washington, USA, a location to which *Coccidioides* spp. are not known to be endemic.

*Coccidioides immitis* is a rare but emerging fungal pathogen in Washington, USA; <5 autochthonous infections are reported annually (*1*). Coccidioidomycosis ranges from asymptomatic infection and mild pulmonary disease to potentially fatal severe disease (*2*,*3*). Infection typically results from inhaling environmental arthroconidia (*3*–*8*).

Only the south-central region of Washington has been established as an area to which *Coccidioides* spp. are endemic; *C. immitis* has been detected in soil from Benton, Yakima, and Kittitas Counties (Figure). In addition, human and canine cases from local exposure have been reported from Franklin, Walla Walla, and Asotin Counties (*8*–*10*). Ecologic models predict a larger endemic area, but no models predict high endemicity in Spokane County (*11*,*12*). We report a possible autochthonous coccidioidomycosis case from Spokane.

**Figure:**
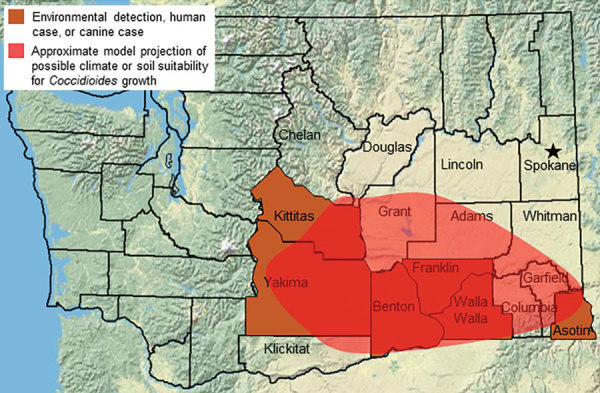
Known and suspected range of *Coccidioides immitis* in Washington, USA. Star indicates residence of case-patient with suspected locally acquired coccidioidomycosis.

## The Study

An 87-year-old woman came to an urgent care center because of a 4-day history of productive cough, fatigue, chest pain, and dyspnea. Chest radiography showed right middle lobe pneumonia; levofloxacin was prescribed without further testing. Four days later (day 8 from symptom onset), she had worsening symptoms and an erythematous maculopapular rash. Antimicrobial drug therapy was switched to concurrent courses of azithromycin for 5 days and cephalexin for 10 days. Repeat chest radiography showed worsening pneumonia and a new pleural effusion. Follow-up visits documented persistent fatigue and productive cough. On day 49, repeat chest radiography showed new bilateral reticular opacities with mild peribronchial wall thickening but no evidence of pleural effusion or worsening infiltrates; a 7-day course of doxycycline was prescribed. Clinical improvement was noted 2 weeks later (day 66): there was resolution of cough, dyspnea, and chest pain.

On day 80, she again had 4 days of congestion and productive cough and received a 5-day course of azithromycin. On day 89, she reported dyspnea, decreased appetite, and weight loss; an albuterol inhaler was prescribed. Three weeks later, she was given a diagnosis of a rectal adenocarcinoma detected by colonoscopy. Positron emission tomography scan (day 109) identified hypermetabolic activity, indicating a right lung mass, which was diagnosed as lung metastasis. A lung biopsy specimen of this mass (day 127) showed no malignant cells but documented spherules morphologically consistent with *Coccidioides* spp.

On day 137, an infectious disease consultant collected serum and prescribed fluconazole, noting pneumonia and 5 months of persistent symptoms consistent with coccidioidomycosis. Results of tests performed at the University of California Davis Serology Laboratory (Davis, CA, USA) (day 146) were positive for IgG and IgM by immunodiffusion and negative by complement fixation; these results were interpreted as indicating a primary coccidioidal infection not well focalized.

Medical records for the patient indicated no recent travel to *Coccidioides* spp.–endemic areas, including south-central Washington. Three months of fluconazole therapy were completed; 13 months after her diagnosis, complement fixation remained negative and symptoms had resolved.

The case-patient was reported to Spokane Regional Health District on day 149. An in-person interview (day 178) elicited lifetime travel history, which included California (2 visits 15–20 years earlier), Oregon (>25 years earlier), and southern Idaho (>3 years earlier). She also reported travel within Washington, including Benton County (>8 years earlier) and Walla Walla County (>8 years earlier). She reported contact with friends who had traveled to Arizona but denied receiving goods from their travel. Her only outdoor activities in the year before onset were nearby walks and scenic drives; she denied gardening or other soil exposures.

Her home was inspected for possible exposure sources, and building employees were interviewed regarding construction and landscaping. No specific exposures were identified; no new landscaping was identified; indoor plants were limited, and central air filters were regularly replaced. Road construction occurred nearby during her exposure period, which could represent a possible infection source.

Record review and patient interview medical history included iliopsoas corticosteroid injections for 2 years before onset; no other immunosuppressing conditions or medications were noted. Records indicated outpatient treatment for pneumonia in 2006 and hospitalization for pneumonia in 2011.

Fixed tissue from lung biopsy specimens was sent to Translational Genomics Research Institute (https://www.tgen.org), where we extracted DNA by using the GeneRead DNA Formalin-Fixed Paraffin-Embedded Kit (QIAGEN, https://www.qiagen.com). We identified *Coccidioides* spp. by using quantitative PCR, as described (*9*,*13*). We performed amplicon sequencing targeting the quantitative PCR region and 10 distinct canonical single-nucleotide polymorphisms (canSNPs) that define the previously described Washington clade (*10*). Sequence reads aligned with the quantitative PCR target at 1,900× depth of coverage, and reads aligned with 2 canSNP targets, at 10× and 6× depth, both suggesting that the sample fits within the Washington *C. immitis* clade. The genomic regions containing the remaining canSNPs had no coverage in these metagenomic samples, limiting a high degree of confidence for true phylogenetic placement.

## Conclusions

We report an apparently autochthonous case of coccidioidomycosis in a woman from Spokane, Washington, a location not known to have *Coccidioides* spp. This patient had many clinical setbacks commonly described with coccidioidomycosis, including 5 unsuccessful antibacterial courses, misdiagnosed lung cancer, and 131 days of healthcare encounters before an accurate diagnosis was obtained.

At least 4 possible hypotheses could explain how this patient became infected. *Coccidioides* infections might be asymptomatic or involve subclinical disease until a reactivation occurs, typically after immunosuppression. First, she could have been infected during travel to south-central Washington (>8 years earlier). Findings of canSNPs matching the known Washington clade support this hypothesis. Second, infection could have occurred during travel to California reported >15 years before onset, although the SNP analysis does not support this hypothesis. The negative complement fixation and positive IgM results suggest more recent infection (*14*), which, along with no calcified lesions observed by chest radiography, would argue against reactivation of infection acquired during previous travel. Third, she could have been exposed through fomite transmission, which has been described (*15*). The patient had visitors who traveled to Arizona; however, no gifts or goods were brought back, and *C. posadasii* would be expected in fomites from Arizona. Plants or soil purchased from another disease-endemic area around her residence are hypothetical sources of exposure, although none were identified. Fourth, it is possible that exposure happened locally in Spokane. Nearby road construction in the weeks before symptom onset could represent a possible source. However, coccidioidomycosis exposures have not been reported from Spokane, and ecologic niche models predict low likelihood of *Coccidioides* habitat (*12*). The nearest known location of detection of *Coccidioides* spp. in soil was ≈130 miles from Spokane. Windborne spores from south-central Washington or through fomite transmission from intrastate commerce or visitors are alternative possibilities. Sequence alignment with 2 canSNP targets and laboratory findings consistent with primary disease support a hypothesis of recent exposure to the Washington clade.

Recent exposure in Spokane County is the most plausible explanation of illness. However, clear documentation of *Coccidioides* spp. endemicity in Spokane will require additional clinical cases or environmental detections.

The environmental range of *Coccidioides* spp. is not fully understood, and is possibly expanding in arid climates (*11*). This possible expansion creates a diagnosis and surveillance challenge because raising and maintaining clinical awareness in low-incidence or emerging settings is difficult. Although determining a definitive exposure source for this case-patient is not possible, the potential for coccidioidomycosis acquisition in new locations cannot be ignored.

Healthcare providers should consider the risk for coccidioidomycosis in patients who reside in or travel to *Coccidioides*-endemic regions (*3*). In addition, healthcare providers should consider fungal infections in patients who have respiratory symptoms that do not respond to antibacterial therapy and should be aware that the geographic risk for coccidioidomycosis is evolving. Ongoing public health surveillance is required to clarify the range of *Coccidioides* spp. and to improve messaging to healthcare providers and the public.
